# Long non-coding RNA (LncRNA) CASC9/microRNA(miR)-590–3p/sine oculis homeobox 1 (SIX1)/NF-κB axis promotes proliferation and migration in breast cancer

**DOI:** 10.1080/21655979.2021.1977555

**Published:** 2021-10-28

**Authors:** Jingzhi Chang, Yuxia Zhang, Xin Ye, Hui Guo, Kun Lu, Qing Liu, Yli Guo

**Affiliations:** Department of Biochemistry and Molecular Biology, Shangqiu Medical College, Shangqiu, China

**Keywords:** NF-κB signaling pathway, CASC9, miR-590-3p, proliferation, breast cancer

## Abstract

Long non-coding RNA (lncRNA)–microRNA–mRNA signaling axes have recently been shown to have a key role in the development of breast cancer (BC). In this study, we investigated how the cancer susceptibility candidate 9 (CASC9) gene affects the cell growth, invasion, migration, and apoptosis of BC cells. The levels of microRNA-590-3p (miR-590-3p), CASC9, and the sine oculis homeobox 1 (SIX1) gene were determined through qRT-PCR. We conducted cell counting kit-8 (CCK-8) assays to assess cell proliferation, transwell assays to detect cell migration/invasion, and flow cytometry to evaluate cell apoptosis. StarBase v2.0 was used to predict interactions between miR-590-3p and SIX1 or CASC9, and dual-luciferase reporter assays were used to verify these predictions. CASC9 protein was overexpressed in BC cells and tissues, while CASC9 knockdown inhibited BC cell growth, invasion, and migration and promoted apoptosis. Additionally, we verified that CASC9 competes for binding with miR-590-3p. Moreover, SIX1 was determined to be a target of miR-590–3p, and SIX1 expression was inhibited by miR-590-3p overexpression. CASC9 enhanced BC development by downregulating miR-590-3p and upregulating SIX1 during the activation of the NF-κB pathway. These data suggest that the CASC9/miR-590-3p/SIX1/NF-κB axis is involved in breast cancer progression, providing insight into the function of CASC9 in breast cancer development.

## 1. Introduction

Breast cancer (BC) is a common malignancy with high incidence in women [1]. Recurrence and metastasis after treatment pose severe threats to survival [2]. BC therapeutics mainly include surgery, chemotherapy, and radiation, but the mechanisms underlying BC development remain unclear [3]. Gene therapy has recently become a hot topic in the fields of cancer diagnosis and treatment. The characterization of tumor suppressor genes and oncogenic genes and their involvement in BC has helped improve the diagnosis and treatment of BC.

Long non-coding RNAs (lncRNAs) are RNAs with a length of over 200 nucleotides that do not encode functional proteins [4]. Several lncRNAs have been reported to participate in tumorigenesis and cancer progression. H19, the first lncRNA discovered, is abnormally expressed in various cancers including BC [5–9]. H19, the Let-7 microRNA (miRNA), and LIN28 form a double-negative feedback loop, which plays a key role in enhancing BC cell growth and differentiation [10]. In addition, NEAT1 is positively correlated with breast cancer stage, such that low NEAT1 levels predict good prognosis whereas high NEAT1 levels predict poor prognosis [11]. The ANCR lncRNA is expressed at low levels in BC cells, which effectively inhibits tumorigenesis and distant metastasis [12]. CDKN2B-AS1 promotes the progression of breast cancer through the miR-122-5p/STK39 axis [13]. CASC9 was reported to promote malignancy in ovarian cancer, colorectal cancer (CRC), lung cancer, esophageal squamous cell carcinoma (ESCC), and BC [14–18]. Although the role of CASC9 in BC development has been studied, the underlying molecular mechanisms have not been fully elucidated.

lncRNAs have been suggested to act as competing endogenous RNAs (ceRNAs) that sponge miRNAs [19]. CASC9 has been reported to act as a ceRNA to target the tumor suppressor miR-125a-3p and regulate neuregulin-1 (NRG1) to promote hemangioma endothelial cell invasion and migration [20]. Moreover, CASC9 positively regulates checkpoint kinase 1 (CHK1) by sponging the miR-195/497 cluster to promote BC cell growth [21]. miR-590 has been identified as a tumor suppressor in different cancers including BC [22–26]. We previously identified miR-590-3p as a downstream target of CASC9 in relation to BC [27].

SIX1 has been identified as an oncogene in various cancers [28–30]. In BC, SIX1 induces the epithelial-mesenchymal transition (EMT) [31]. In addition, SIX1 was found to upregulate vascular endothelial growth factor-C (VEGF-C) expression to induce lymphangiogenesis and metastasis in BC mouse models [32]. Recent data suggest that several miRNAs including miR-362, miR-186-5p, and miR-188 target the 3ʹ untranslated regions (3ʹ UTRs) of the SIX1 mRNA to induce its degradation and inhibit its translation in several cancers [33–35]. Through bioinformatic analysis, we discovered putative miR-590-3p binding sites in the SIX1 3ʹ UTR. Considering that miR-590-3p is a downstream target of CASC9, we reasoned that miR-590–3p might represent the ‘bridge’ for CASC9 and SIX1 in BC. Furthermore, SIX1 overexpression has been previously suggested to activate the NF-κB pathway [36]. Thus, CASC9 promotes BC progression by regulating miR-590-3p and modulating the SIX1/NF-κB axis. The present study explored CASC9 expression in BC cells and tissues, while exploring its roles in BC cell growth, invasion, migration, and apoptosis, and the underlying mechanisms. We hypothesized that the CASC9 lncRNA affects BC cell proliferation, migration, and invasion via the miR-590–3p/SIX1 axis.

## 2. Materials and methods

### 2.1 Patients and sample collection

From January 2018 to December 2019, 42 tissue specimens were collected from patients with BC who underwent BC surgery at Shangqiu Central Hospital (Henan, China). All samples were pathologically confirmed to be without preoperative radiotherapy, chemotherapy, or any other treatment. The tissue samples were immersed in liquid nitrogen after removal. Our experimental procedures were approved by the Research Ethics Committee of Shangqiu Central Hospital. This study was performed in accordance with the Declaration of Helsinki. The participants provided informed consent for participation.

### 2.2 Cell culture

Normal breast epithelial cells (MCF-10A) and BC cells (MDA-MB-468, MCF7, and MDA-MB-231) were provided by the BeNa Culture Collection (Beijing, China) and cultured in high-glucose (HG) DMEM containing 10% fetal bovine serum (FBS). MDA-MB231 and MCF7 cells were cultivated in RPMI 1640 medium containing 10% FBS. Each sample was incubated at 37°C under 5% CO_2_ conditions [37].

### 2.3 Cell line transfection

si-CASC9, miR-590–3p mimics, and corresponding negative controls (NCs) were obtained from GenePharma (Shanghai, China). Cells (6 × 10^5^/well) were seeded into 6-well plates and the above plasmids, a pcDNA3.1 empty vector, and a pcDNA3.1-SIX1 vector were separately transfected into MDA-MB231 and MCF7 cells using Lipofectamine 2000 Reagent (Invitrogen, Carlsbad, CA, USA). following previously established protocols [27].

### 2.4 Quantitative real-time PCR (qRT-PCR)

Total RNA was isolated using TRIzol™ Reagent (Invitrogen, Carlsbad, CA, USA) and quantified. The PrimeScript™ RT Kit (Takara, Dalian,China) was used to reverse transcribe 1 µg of RNA into cDNA. SYBR Green PCR Master Mix Kit (Invitrogen, Thermo Fisher Scientific, Inc.; cat. no. 4,309,155) was used in qRT-PCR reactions run on the ABI 7500HT Real-Time PCR machine (Takara Bio, Shiga, Japan) according to established protocols [27]. The thermocycling conditions were as follows: 10 min at 94°C followed by 40 cycles of 30 s at 94°C, 34 s at 54°C, and 30 s at 72°C. The 2^−ΔΔCt^ approach was utilized to calculate relative transcript levels, with *U6* and *GAPDH* serving as reference genes for miR-590-3p and CASC9/SIX1, respectively. The sequences of all primers used in this study are presented in Table 1.

**Table 1.** The primer sequences

Gene	Position	Sequence (5`to3`)
CASC9	Forward	CAGGTAATCTCAGCAGTCAT
	Reverse	ACATCCACAGGTCTCCAA
GAPDH	Forward	CAAGGTCATCCATGACAACTTTG
	Reverse	GTCCACCACCCTGTTGCTGTAG
miR-590-3p	Forward	TAATTTTATGTATAAGCTAGT
	Reverse	GCAGGGTCCGAGGTATTC
U6	Forward	TCCGATCGTGAAGCGTTC
	Reverse	GTGCAGGGTCCGAGGT
SIX1	Forward	AAGGTGAGTGGTGTATTGC
	Reverse	GAATGCTGTGAAGAGATAGTG
si-CASC9^1#^		AUGAACAUCCACAAACACCAA
si-CASC9^2#^		UAAUAUUUCUUGAUAGUGCCA
si-CASC9 NC		GAAUCCUACUUUCACAGCCAU
miRNA-590-3p mimics miRNA-590-3p mimics NC si-SIX1 si-SIX1 NC		TAATTTTATGTATAAGCTAGT CTAGTCACTATATAGGAGCTG GCUUGUUUCUGGAGUUGUUUG UCCUUUAGGGUGCGAUGGUUU

### 2.5 Cell Counting Kit-8 (CCK-8) assay

A CCK-8 assay was conducted to measure cell proliferation. In brief, after digestion, transfected MCF7 and MDA-MB231 cells (3 × 10^5^/well) were inoculated into 96-well plates and incubated under 5% CO_2_ at 37°C for 1, 2, 3, 4, and 5 days. After adding 10 µL CCK-8 solution (Dojindo Chemical, Japan), the cells were cultured for 2 h. Finally, the optical density (OD) was determined at 490 nm and the absorbance was measured in triplicate [38].

### 2.6 Cell migration and invasion assay

Transfected cells (1.0 × 10^6^) were prepared in a cell suspension and added to the top of a Transwell chamber filled with serum-free medium (1 mL), while the bottom chamber was filled with 10% FBS in 600 µL DMEM (Sangon Biotech Co., Ltd.). After 24 h, cells in the top chamber were collected and subjected to 95% ethanol fixation, 15 min of 0.05% crystal violet staining, and observation and enumeration using an optical microscope (Nikon, Tokyo, Japan) at 100× magnification. For the invasion assays, transwell chambers were coated with Matrigel (Millipore, USA) [38].

### 2.7 Western blotting (WB)

ter 24 h of transfection, MCF7 and MDA-MB231 cells were collected and lysed in RIPA buffer to isolate total protein, which was quantified using a BCA assay (Beyotime Biotechnology, Shanghai, China). Next, total protein samples (30 µg) were separated on a 10% SDS-PAGE gel, followed by the transfer of the proteins to PVDF membranes (Beyotime Biotechnology, Shanghai, China). Membranes were incubated with primary antibodies overnight at 4°C, followed by incubation with secondary antibodies for 2 h at room temperature. Membranes were washed with TBST, developed using ECL and a gel imaging system (Bio-Rad) and assessed by One software (Bio-Rad, USA) [38].

### 2.8 Luciferase reporter assay

The pmirGLO Dual-Luciferase Reporter Gene Detection System (Promega, Madison, WI, USA) was used to synthesize SIX1- and CASC9-binding sites for miR-590-3p in the corresponding 3ʹ UTR. These constructs were inserted into the pmirGLO luciferase expression vector to generate a pmirGLO/CASC9 vector and a pmirGLO/SIX1-UTR vector. The mutant sequence was synthesized to generate the pmirGLO/CASC9-M and pmirGLO/SIX1-M-UTR vectors using the same method. We used Lipofectamine 2000 (Invitrogen, Thermo Fisher Scientific, Inc.) to transfect the pmirGLO/CASC9 and pmirGLO/SIX1-UTR wild-type vectors, pmirGLO/CASC9-M, pmirGLO/SIX1-M-UTR vectors, and miR-590–3p mimics, together with the corresponding NC, into MCF7 and MDA-MB231. The cells were harvested 48 h after transfection. The fluorescence value of the sea kidney served as the endogenous control, as suggested by the manufacturer. The fluorescence ratio (firefly to sea kidney) was determined to evaluate the relative activity of the reporter gene [27].

### 2.9 Construction of the pCDH-Flag-SIX1 recombinant plasmid

SIX1 was amplified for 34 cycles (5 min of pre-denaturation at 95°C, 30 s of denaturation at 95°C, 30 s of annealing at 62°C, 60 s of extension at 72°C, and 7 min of extension at 72°C). PCR products were visualized by gel electrophoresis, and Not *I* and Bam*HI* were used to digest the amplicons and the pCDH-Flag vector. T4 DNA ligase was used to ligate the amplicons into the pCDH-flag vector at 16°C for 30 minutes. The resulting vectors were transformed into *Escherichia coli* DH5α cells and cultured on LB plates containing 100 μg/ml ampicillin [39].

### 2.10 Flow cytometry

Transfected cells were digested using trypsin, centrifuged, and harvested. Cells were rinsed twice with PBS, resuspended in 70% ethanol, and fixed at 4°C for 30 min. Next, the fixed cells were washed twice with PBS and incubated in AnnexinV-FITC and propidium iodide (PI, Jiancheng, Nanjing, China) for 1 h at 4°C, at which point quantifications were performed [40].

### 2.11 Bioinformatic analysis

We searched the GEPIA database (http://gepia.cancer- pku.cn/) to analyze SIX1 expression in BC samples [41]. The LncBase Predicted v.2 (http://carolina.imis.athena-innovation.gr/diana_tools/web/index.php?r=lncbasev2%2Findex-predicted) [42] and TargetScan 7.2 databases (http://www.targetscan.org/vert_72/) [43] were used to search for miR-590-3p binding sites in CASC9 and the SIX1 3ʹ UTR. Regarding the search for miR-590-3p binding sites, the LncBase Predicted v.2 database was used to input the location of CASC9 (chr8:75,120,409–75,352,327) and then identify binding sites for miRNAs, including miR-590-3p.

### 2.12 Statistical analysis

SPSS24.0 software was used to perform all statistical analyses. Data are displayed as the mean ± standard deviation (SD). Significant differences between groups were identified using Student’s *t*-tests or ANOVA followed by Tukey’s post-hoc test. Correlations were determined using Pearson’s correlation analysis. Statistical significance was set at *p*< 0.05.

## 3. Results

In this study, we explored the role of CASC9 in BC. A series of assays led to the discovery that CASC9 promotes BC progression by regulating the miR-590-3p/SIX1/NF-κB axis. Our findings highlight the functional roles of CASC9 in BC, providing new insights into BC pathogenesis.

### 3.1 CASC9 is upregulated in BC tissues and cells

**Figure 1.**
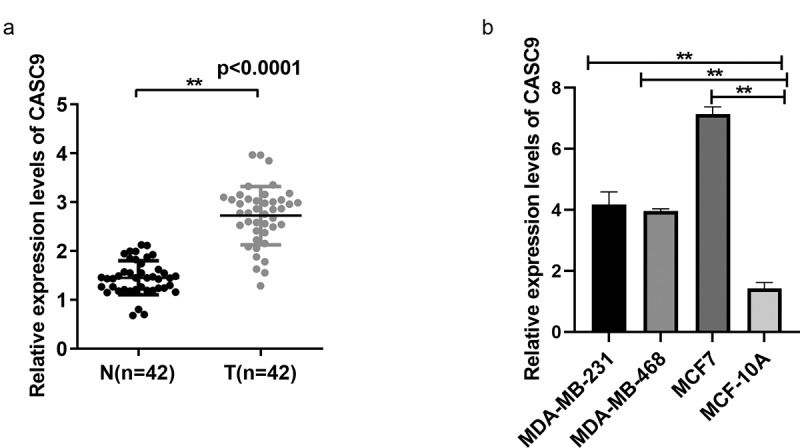
CASC9 significantly up-regulated within BC cells and tissues

We first interrogated CASC9 expression in 42 pairs of BC and non-carcinoma tissues by qRT-PCR. CASC9 levels were significantly increased in BC samples compared to the levels in control samples (Figure 1a). CASC9 levels were examined in normal breast epithelial MCF-10A cells and BC cells (MDA-MB-231, MDA-MB-468, and MCF7) by qRT-PCR. CASC9 was found to be highly expressed in MDA-MB231, MCF7, and MDA-MB-468 cell lines (Figure 1b), suggesting that CASC9 is closely related to BC progression. CASC9 expression was the highest in the MCF7 and MDA-MB231 cell lines, therefore these cell lines were selected for the subsequent experiments.

(A) CASC9 level in 42 BC tissues relative to non-carcinoma samples. (B) CASC9 expression level within BC cells (MDA-MB-468, MCF-7, MDA-MB-231) and MCF-10A cells. **P< 0.01.

### 3.2 CASC9 knockdown suppresses BC cell proliferation and promotes apoptosis

**Figure 2.**
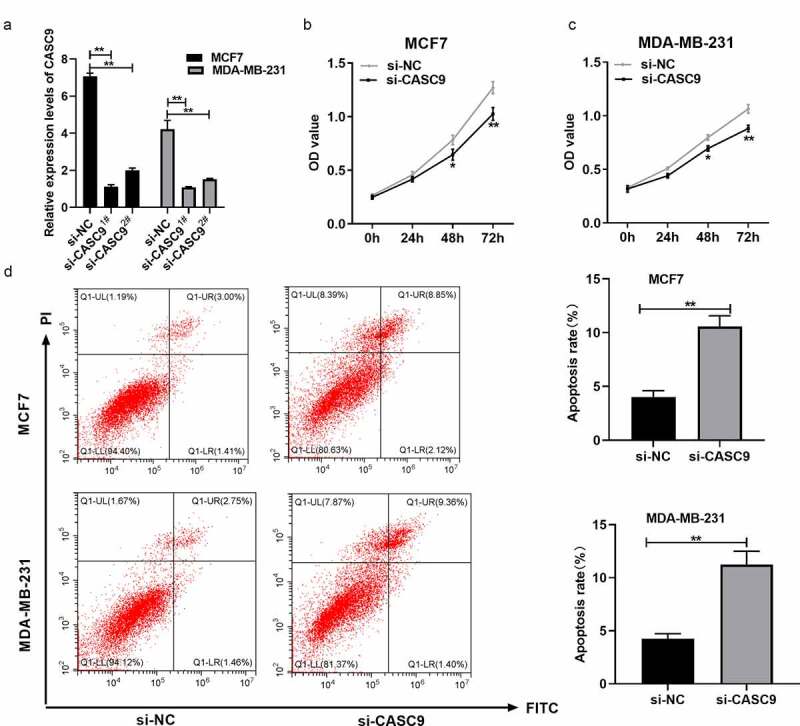
Knock-down of CASC9 inhibits BC cell growth but promotes their apoptosis

siRNA-CASC9 was used to downregulate CASC9, and qRT-PCR was used to confirm that CASC9 expression was lower in the siRNA-CASC9^1#^ group than in the siRNA-CASC9^2#^ group (Figure 2a). Therefore, siRNA-CASC9^1#^ was used for further experiments. CCK8 and flow cytometry assays were used to investigate the function of CASC9 in BC development. CASC9 knockdown significantly reduced BC cell proliferation (Figure 2b and c) and increased apoptosis in MCF7 and MDA-MB231 cells (Figure 2d). These results suggest that CASC9 promotes BC cell proliferation and inhibits cell apoptosis.

(A) CASC9 levels within MDA-MB-231 and MCF7 cells subjected to si-CASC9- or si-NC transfection were analyzed via qRT-PCR. (B) CCK-8 assay analysis of cell proliferation abilities in MCF-7 cells subjected to si-CASC9 or si-NC transfection. (C) CCK-8 assay was conducted for detecting MDA-MB-231 cell proliferation subjected to si-CASC9 or si-NC transfection. (D) Flow cytometry analysis of cell apoptosis abilities of MCF-7 and MDA-MB-231 cells subjected to si-CASC9- or si-NC transfection. *P< 0.05, **P< 0.01.

### 3.3 CASC9 knockdown suppresses BC cell migration and invasion

**Figure 3.**
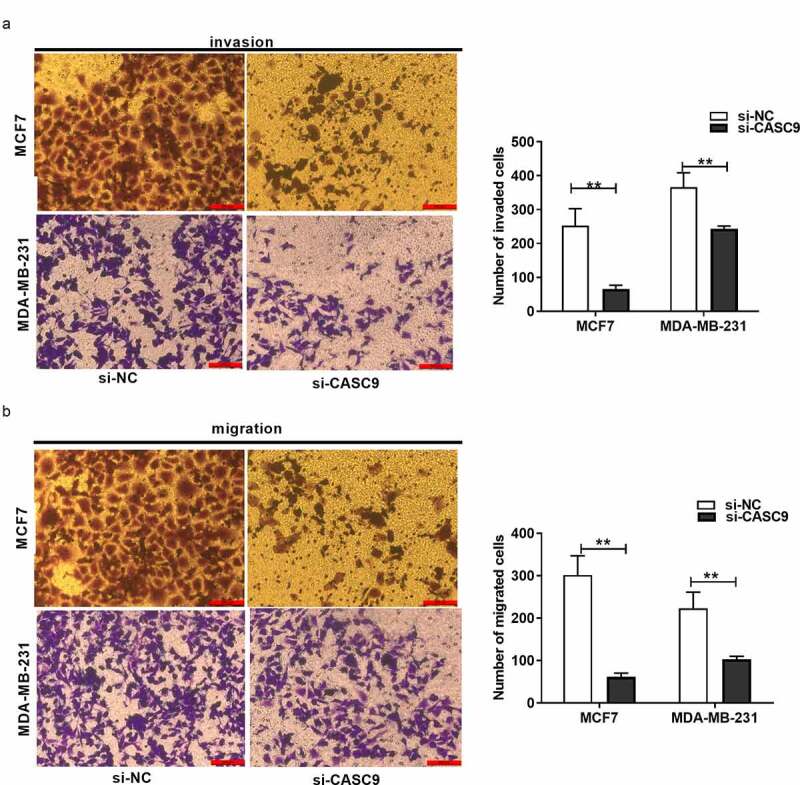
Knock-down of CASC9 suppresses breast cancer cell invasion and migration

Transwell migration and invasion experiments revealed that, compared to the NC group, the number of invading and migrating BC cells markedly decreased in response to CASC9 knockdown (Figure 3a and b), suggesting that CASC9 promotes BC cell migration and invasion.

(A) Transwell assays analysis of MDA-MB-231 and MCF-7 cell migration after si-CASC9- or si-NC transfection. (B) Transwell assays analysis on the invasion of the above two cell lines after si-CASC9- or si-NC transfection. **P< 0.01.

### 3.4 miR-590-3p is a target of CASC9

**Figure 4.**
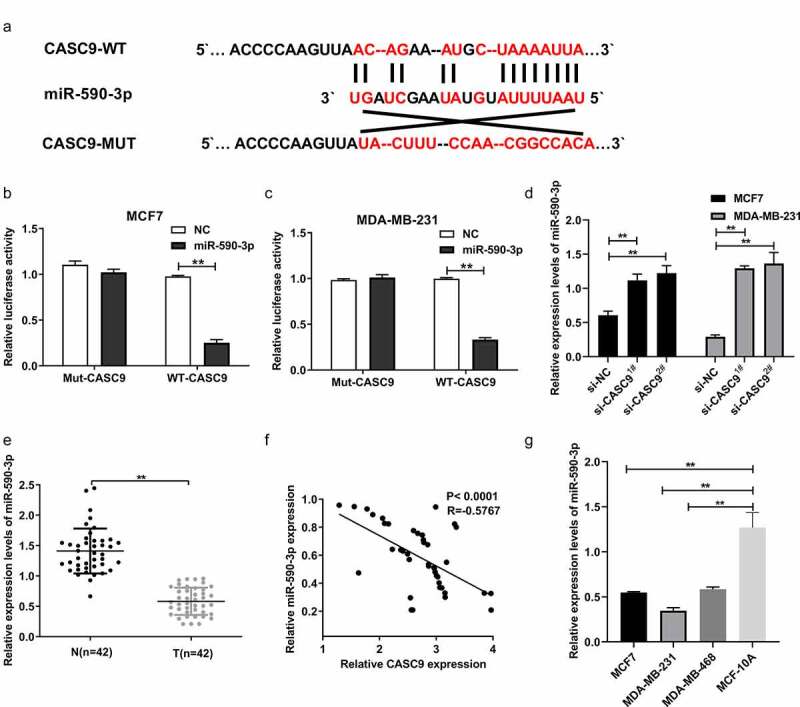
miR-590-3p as a CASC9 candidate target

To further explore the molecular mechanism underlying the role of CASC9 in the development and progression of BC, putative CASC9-binding sites were predicted using LncBase v.2. Interestingly, bioinformatic analysis revealed that miR-590-3p was a potential target of CASC9 (Figure 4a). A physical interaction between miR-590-3p and CASC9 was observed in MDA-MB231 and MCF7 cells using a luciferase reporter assay (Figure 4b and c). In addition, we revealed that knockdown of CASC9 markedly upregulated miR-590-3p expression in MCF7 and MDA-MB231 cells relative to the NC (Figure 4d). We also measured miR-590-3p expression in BC tissues and cells, and found that miR-590-3p expression was decreased in BC cells and tissues in a manner that was directly proportional to CASC9 levels in BC tissue samples (Figure 4e-g).

(A) miR-590-3p binding sites for CASC9. (B) CASC9 mut or WT luciferase reporter plasmid was co-transfected in MCF-7 cells with miR-590-3p mimics or corresponding NC for luciferase assays. (C) CASC9 mut or WT luciferase reporter plasmid was co-transfected into MDA-MB-231 cells with miR-590-3p mimics or corresponding NC for luciferase assays. (D) miR-590-3p levels in MDA-MB-231 and MCF-7 cell lines subjected to si-CASC9 or si-NC transfection. (E) miR-590-3p levels within 42 BC tissue samples relative to matched non-carcinoma samples. (F) Correlation of CASC9 levels with miR-590-3p levels within BC tissues. (G) miR-590-3p levels within normal breat epithelial MCF-10A cells and BC cells (MDA-MB-231, MDA-MB-468, MCF7). **P< 0.01.

### 3.5 CASC9 promotes malignancy of BC cells by decreasing miR-590-3p levels

**Figure 5.**
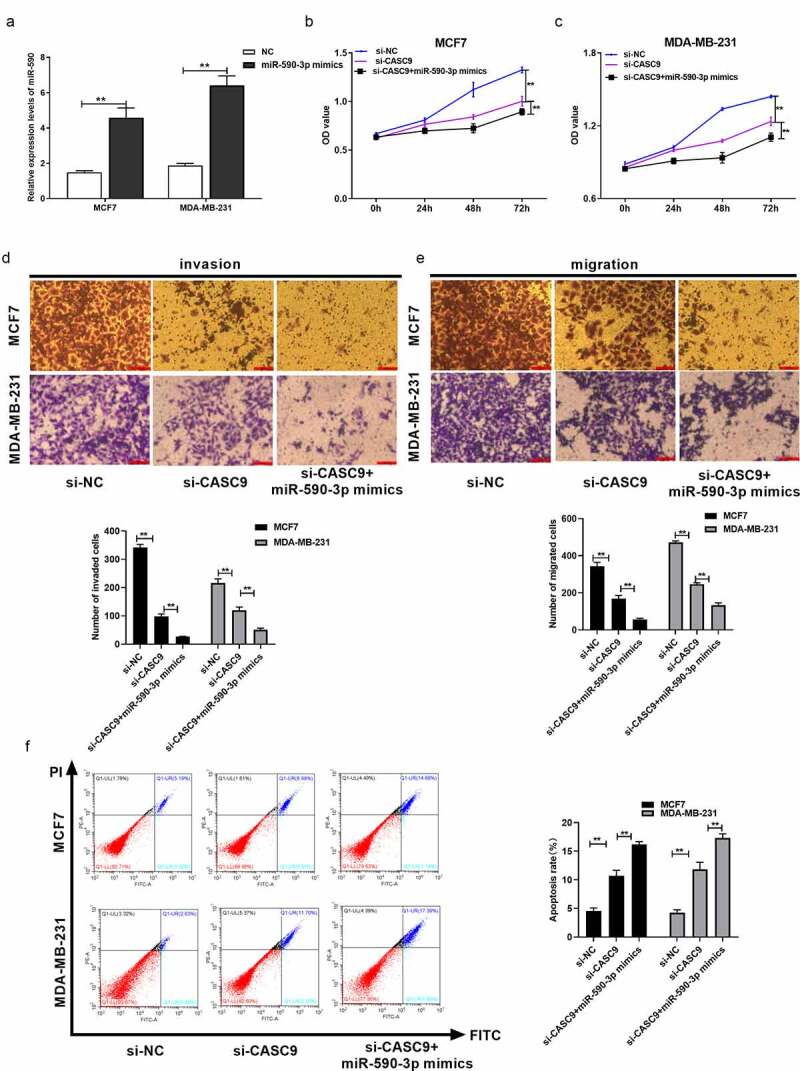
CASC9 promotes malignant behavior of BC cells through reducing miR-590-3p

To further explore whether CASC9 promoted malignancy by regulating miR-590-3p in BC, we co-transfected si-CASC9 and miR-590-3p mimics into BC cells. First, miR-590-3p mimics, together with the corresponding NCs, were transfected into MCF7 and MDA-MB231 cells to determine the transfection efficiency. The miR-590-3p mimics group was characterized by remarkably higher miR-590-3p mRNA levels compared to the NC group, demonstrating successful transfection (Figure 5a). In addition, we investigated whether CASC9 promotes the malignancy of breast cancer cells by interacting with miR-590-3p. To verify this hypothesis, we transfected miR-590-3p mimics and si-CASC9 in MCF7 and MDA-MB231 cells. We confirmed that CASC9 knockdown remarkably suppressed cell growth, invasion, and migration, but enhanced apoptosis. However, these effects were enhanced when cells were co-transfected with miR-590-3p mimics (Figure 5b-f). Based on these findings, CASC9 may promote the malignancy of BC cells by decreasing miR-590-3p.

(A) miR-590-3p levels within MDA-MB-231 and MCF7 cells subjected to miR-590-3p mimics or mimics-NC transfection were measured via qRT-PCR assay. (B) MCF-6 cell proliferation subjected to miR-590-3p mimics (or NC) and/or si-CASC9 (or si-NC) transfection detected via CCK-8 assay. (C) MDA-MB-231 cell proliferation subjected to miR-590-3p mimics (or NC) and/or si-CASC9 (or si-NC) transfection measured by CCK-8 assay. (D) Transwell assays of MDA-MB-231 and MCF7 cell migration following miR-590-3p mimics (or NC) and/or si-CASC9 (or si-NC) transfection. (E) Transwell assays analysis of cell invaded abilities within MDA-MB-231 and MCF7 cells subjected to miR-590-3p mimics (or NC) and/or si-CASC9 (or si-NC) transfection. (F) Flow cytometry analysis of cell apoptosis abilities within the above two cell lines following miR-590-3p mimics (or NC) and/or si-CASC9 (or si-NC) transfection. **P< 0.01.

### 3.6 SIX1 is a direct target of miR-590-3p in BC cells

**Figure 6.**
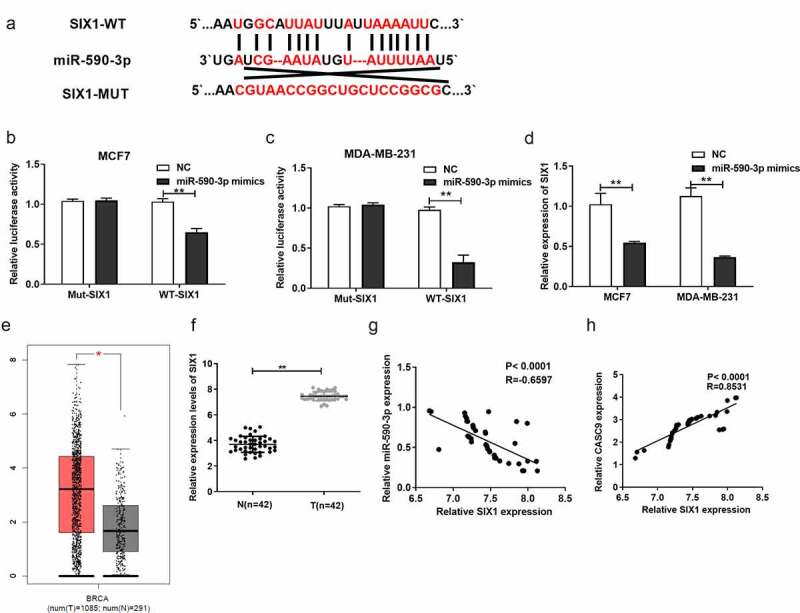
SIX1, a direct miR-590-3p target within BC cells

TargetScan analyses predicted that SIX1 may be a direct target of miR-590-3p (Figure 6a). Luciferase reporter assays verified that miR-590-3p directly interacted with CASC9 in MCF7 and MDA-MB231 cells (Figure 6b and c). In addition, qRT-PCR assays validated that miR-590-3p overexpression markedly decreased SIX1 expression (Figure 6d). Additionally, SIX1 levels were increased in BC patient tumor samples compared to normal samples, as evidenced by the analysis of The Cancer Genome Atlas database using GEPIA (Figure 6e). SIX1 was highly expressed in BC tissues (figure 6f) in a manner that was indirectly proportional to miR-590-3p expression (Figure 6g) and positively correlated with CASC9 levels in BC tissue samples (Figure 6h). These findings suggest that SIX1 is directly targeted by miR-590-3p.

(A) SIX1 binding sites in miR-590-3p. (B) SIX1 mut or WT luciferase reporter plasmid was co-transfected in MCF-7 cells with miR-590-3p mimics or corresponding NC for luciferase assays. (C) SIX1 mut or WT luciferase reporter plasmid was co-transfected into MDA-MB-231 cells with miR-590-3p mimics or corresponding NC for luciferase assays. (D) SIX1 levels within MCF-7 and MDA-MB-231 cells after NC or miR-590-3p mimics transfection. (E) The expression level of SIX1 in BC tumor samples and normal samples predicted by GEPIA website. (F) SIX1 expression within 42 BC samples relative to matched non-carcinoma samples. (G) Correlation of SIX1 level with miR-590-3p level within BC tissues. (H) The correlation analysis between SIX1 expression and CASC9 level within BC samples. **P< 0.01.

### 3.7 CASC9 promotes the malignancy of BC cells by decreasing miR-590-3p levels and upregulating SIX1

**Figure 7.**
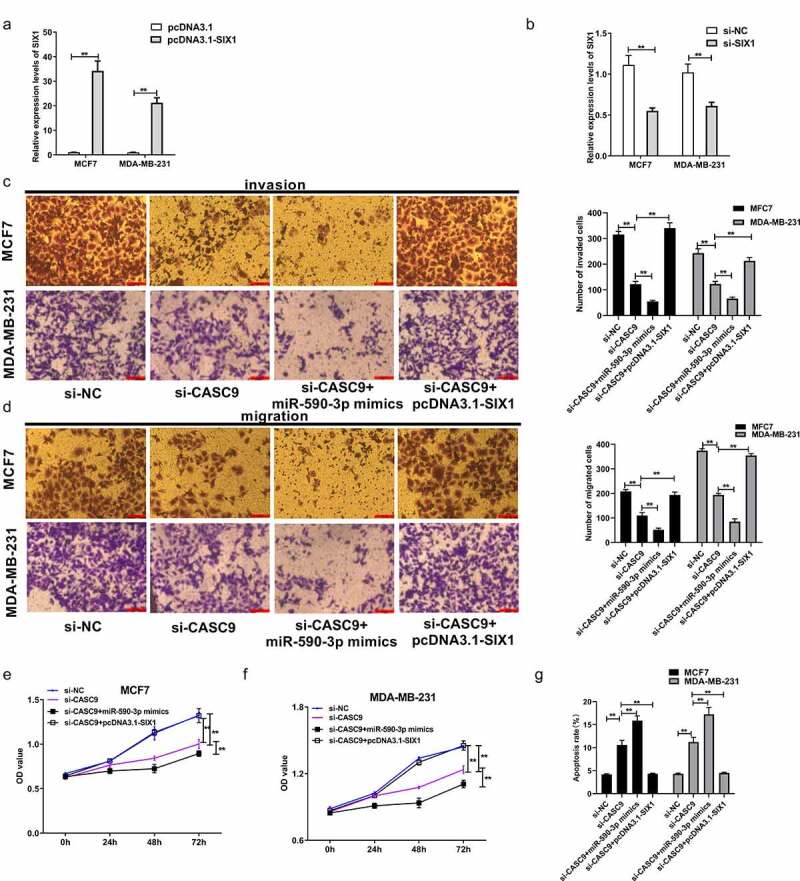
CASC9 promotes malignant behavior of BC cells by down-regulating miR-590-3p and up-regulating SIX1

As previously mentioned, CASC9 binds to miR-590-3p, while miR-590-3p targets SIX1 to exert its function in BC cells. To further explore whether CASC9 promoted the malignancy of BC cells by regulating the miR-590-3p/SIX1 axis in BC, si-SIX1 (si-NC) and pcDNA-SIX1 (pcDNA3.1) were transfected into MCF7 and MDA-MB231 cells. qRT-PCR analysis demonstrated that SIX1 mRNA levels were decreased in response to si-SIX1, while SIX1 mRNA levels increased in response to pcDNA-SIX1, indicating successful transfection (Figure 7a and b). A series of functional assays demonstrated that transfection of si-CASC9 markedly suppressed BC cell proliferation, migration, and invasion, which markedly increased after miR-590-3p mimic co-transfection, while co-transfection with pcDNA-SIX1 restored these effects (Figure 7c-f). Moreover, CASC9 knockdown increased BC cell apoptosis, which was markedly increased by co-transfection of a miR-590-3p mimic, an effect that was abolished by SIX1 overexpression (Figure 7g). These observations suggest that CASC9 enhances BC malignancy by decreasing miR-590-3p expression and upregulating SIX1.

(A) SIX1 levels within pcDNA3.1-SIX1- or pcDNA3.1-transfected MDA-MB-231 and MCF7 cell lines measured through qRT-PCR. (B) SIX1 levels within MDA-MB-231 and MCF7 cells following si-SIX1 or si-NC transfection measured by qRT-PCR. (C) Transwell assays analysis of migration abilities within the above two cell lines. (D) Transwell assays analysis of invaded abilities within the above two cell lines. (E) Viability of MCF7 cells measured through CCK-8 assay. (F) Viability of MDA-MB-231 cells detected through CCK-8 assay. (G) MDA-MB-231 and MCF7 cell apoptosis examined through flow cytometry. **P< 0.01.

### 3.8 The CASC9/miR-590-3p/SIX1/NF-κB axis is involved in BC progression

**Figure 8.**
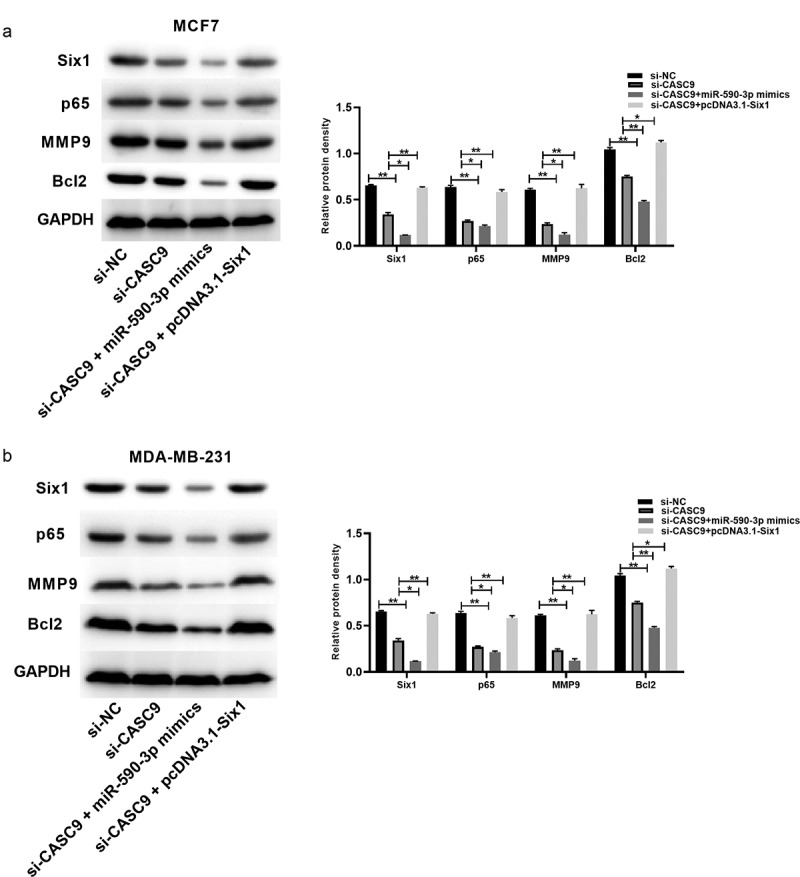
CASC9/miR-590-3p/SIX1/NF-κB pathway related to BC progression

SIX1 silencing was reported to inhibit NF-κB activation [36]. We discovered that CASC9 knockdown decreased the levels of related proteins such as SIX1, p65, matrix metalloproteinase 9 (MMP9), and B-cell lymphoma 2 (BCL2), while miR-590-3p overexpression enhanced these effects and SIX1 overexpression reversed these effects (Figure 8a-b). These findings confirm that the CASC9/miR-590-3p/SIX1/NF-κB axis is involved in BC progression.

(A) Protein levels within MCF7 cells detected through WB assay. (B) Protein levels within MDA-MB-231 cells detected through WB assay. All bars stand for mean ± SD for 3 individual assays. *P< 0.05, **P< 0.01.

## 4. Discussion

lncRNAs are endogenous, single-stranded, non-coding RNAs with lengths of over 200 nucleotides [4]. They participate in epigenetic regulation via chromatin remodeling, transcriptional regulation, and post-transcriptional regulation [44]. lncRNAs also function in regulating cell growth, differentiation, neural development, immune homeostasis, tumorigenesis, and other biological processes [45–49]. Several studies have examined the functions of lncRNAs in BC [8,11,13]. For instance, high expression of the VCAN-AS1 lncRNA was detected in BC, which promotes the malignancy of BC by regulating the miR-106a-5p-regulated signal transducer and activator of transcription 3/hypoxia-inducible factor-1alpha pathway [37]. The BCAR4 lncRNA is overexpressed in BC cells and tissues, which promotes BC cell proliferation by suppressing p16 expression [50]. The CASC9 oncogene is highly expressed in esophageal squamous cell carcinoma (ESCC), ovarian cancer, BC, lung cancer, papillary thyroid cancer, and bladder cancer [14–17,51,52]. The CASC9 lncRNA positively regulates CHK1 to promote the growth and survival of BC cells by sponging the miR-195/497 cluster. Zhang et al. showed that CASC9 promotes BC cell metastasis by mediating the miR-215/Twist-related protein 2 signal transduction pathway in BC [53]. Shao et al. reported that CASC9 enhanced the growth and survival of BC cells by sponging the miR-195/497 cluster [21]. However, the effect of CASC9 in BC had not yet been investigated. Understanding its role in BC may shed light on the development and progression of BC.

The present study discovered that the CASC9 lncRNA was highly expressed in BC tissues and cells, indicating a possible regulatory role for CASC9 in BC progression. To explore the role of CASC9 in BC, we silenced CASC9 in MCF7 and MDA-MB231 BC cells. Furthermore, we demonstrated that low expression of CASC9 remarkably suppressed cell growth, migration, and invasion, but enhanced apoptosis.

lncRNAs can act as molecular sponges by binding to miRNAs to inhibit the miRNA-mediated silencing of target mRNAs. TargetScan analysis indicated that miR-590-3p might be a binding partner of CASC9. Bioinformatic predictions and dual-luciferase reporter assays revealed that SIX1 is a target of miR-590-3p. Thus, the upregulation of SIX1 in BC tissues was confirmed. Thus, in this study, we characterized the CASC9/miR-590-3p/SIX1 regulatory network.

The NF-κB pathway is essential to immunity, and its importance in cancer development has been extensively studied, especially in relation to tumor cell proliferation, survival, and metastasis. Multiple studies have shown that lncRNAs and several protein-coding genes regulate the NF-κB signaling pathway [54–56]. As reported by Yang et al., SIX1 knockdown dramatically suppressed NF-κB activation [36]. Han et al. reported that the DNAJC3-AS1/miR-214-3p axis affected NF-κB activation via the regulation of LIVIN expression, which further impacted the malignant phenotype of colorectal cancer cells [56]. Gao et al. demonstrated that the inhibition of XIST promoted U2OS cell death via the activation of the NF-κB/PUMA pathway [55]. Here, we examined the levels of the NF-κB protein p65, apoptosis-associated protein Bcl2, and MMP9. SIX1, p65, MMP9, and Bcl2 protein levels were reduced by CASC9 knockdown, and miR-590-3p overexpression enhanced these effects. Thus, the CASC9/miR-590-3p/SIX1/NF-κB axis is involved in BC progression. However, there are some limitations to our study. For example, an electrophoretic mobility shift assay was not performed to confirm the involvement of the NF-κB signaling pathway. In addition, RNA immunoprecipitation and RNA pull-down assays were not performed to confirm the relationship between CASC9, miR-590-3p, and SIX1.

## 5. Conclusion

In this study, we established that CASC9 regulates BC tumorigenesis by modulating the SIX1/NF-κB signaling pathway via miR-590-3p. Thus, CASC9 may represent an attractive therapeutic target for the prevention and treatment of BC.

## Data Availability

The data used to support the findings of this study are available from the corresponding author upon request.

## Highlights

(1) CASC9 was up-regulated in BC.
(2) CASC9 promoted proliferation, migration, invasion and inhibited apoptosis of BC cells.
(3) CASC9 regulated SIX1 by sponging miR-590-3p.
(4) CASC9/miR-590-3p/SIX1 regulated BC through NF-κB signaling.

## References

[1] Bertucci F, Ng CKY, Patsouris A, et al. Genomic characterization of metastatic breast cancers. Nature. 2019;569(7757):560–564. 31118521 10.1038/s41586-019-1056-z

[2] Siegel RL, Miller KD, Jemal A. Cancer statistics, 2019. CA Cancer J Clin. 2019;69(1):7–34. 30620402 10.3322/caac.21551

[3] Nagini S. Breast cancer: current molecular therapeutic targets and new players. Anticancer Agents Med Chem. 2017;17(2):152–163. 27137076 10.2174/1871520616666160502122724

[4] Ponting CP, Oliver PL, Reik W. Evolution and functions of long noncoding RNAs. Cell. 2009;136(4):629–641. 19239885 10.1016/j.cell.2009.02.006

[5] Zhao W, Lin X, Han H, et al. Long noncoding RNA H19 contributes to the proliferation and autophagy of glioma cells through mTOR/ULK1 pathway. Neuroreport. 2021;32(5):352–358. 33661803 10.1097/WNR.0000000000001602

[6] Zhang W, Zhou K, Zhang X, et al. Roles of the H19/microRNA‑675 axis in the proliferation and epithelial‑mesenchymal transition of human cutaneous squamous cell carcinoma cells. Oncol Rep. 2021;45(4):1–13. 33649811 10.3892/or.2021.7990 PMC7905556

[7] Wang Y, Zhou P, Li P, et al. Long non-coding RNA H19 regulates proliferation and doxorubicin resistance in MCF-7 cells by targeting PARP1. Bioengineered. 2020;11(1):536–546. 32345117 10.1080/21655979.2020.1761512 PMC8291873

[8] Li Y, Ma HY, Hu XW, et al. LncRNA H19 promotes triple-negative breast cancer cells invasion and metastasis through the p53/TNFAIP8 pathway. Cancer Cell Int. 2020;20(1):200. 32514245 10.1186/s12935-020-01261-4 PMC7257135

[9] Yan L, Yang S, Yue C-X, et al. Long noncoding RNA H19 acts as a miR −340-3p sponge to promote epithelial-mesenchymal transition by regulating YWHAZ expression in paclitaxel-resistant breast cancer cells. Environ Toxicol. 2020;35(9):1015–1028. 32420678 10.1002/tox.22938

[10] Xiong H, Shen J, Chen Z, et al. H19/let‑7/Lin28 ceRNA network mediates autophagy inhibiting epithelial‑mesenchymal transition in breast cancer. Int J Oncol. 2020;56(3):794–806. 32124962 10.3892/ijo.2020.4967

[11] Huang Y, Wang X, Zheng Y, et al. Construction of an mRNA-miRNA-lncRNA network prognostic for triple-negative breast cancer. Aging (Albany NY). 2021;13(1):1153–1175. 33428596 10.18632/aging.202254 PMC7835059

[12] Wang S, Lan F, Xia Y. lncRA ANCR inhibits non-small cell lung cancer cell migration and invasion by inactivating TGF-β pathway. Med Sci Monit. 2018;24:6002–6009. 30154397 10.12659/MSM.911492 PMC6126415

[13] Qin S, Ning M, Liu Q, et al. Knockdown of long non-coding RNA CDKN2B-AS1 suppresses the progression of breast cancer by miR-122-5p/STK39 axis. Bioengineered. 2021;12(1):5125–5137. 34374638 10.1080/21655979.2021.1962685 PMC8806778

[14] Hu X, Li Y, Kong D, et al. Long noncoding RNA CASC9 promotes LIN7A expression via miR-758-3p to facilitate the malignancy of ovarian cancer. J Cell Physiol. 2019;234(7):10800–10808. 30537154 10.1002/jcp.27903

[15] Liang Y, Chen X, Wu Y, et al. LncRNA CASC9 promotes esophageal squamous cell carcinoma metastasis through upregulating LAMC2 expression by interacting with the CREB-binding protein. Cell Death Differ. 2018;25(11):1980–1995. 29511340 10.1038/s41418-018-0084-9 PMC6219493

[16] Zhao W, Chen T, Zhao Y. Upregulated lncRNA CASC9 contributes to progression of non-small cell lung cancer through inhibition of miR-335-3p and activation S100A14 Expression. Onco Targets Ther. 2020;569:6027–6036. 10.2147/OTT.S249973 PMC7321690 32606808

[17] Jiang B, Li Y, Qu X, et al. Long noncoding RNA cancer susceptibility candidate 9 promotes doxorubicin‑resistant breast cancer by binding to enhancer of zeste homolog 2. Int J Mol Med. 2018;42(5):2801–2810. 30106089 10.3892/ijmm.2018.3812

[18] Liu H-Z, Shan T-D, Han Y, et al. Silencing long non-coding RNA CASC9 inhibits colorectal cancer cell proliferation by acting as a competing endogenous RNA of miR-576-5p to regulate AKT3. Cell Death Discov. 2020;6(1):115. 33298846 10.1038/s41420-020-00352-5 PMC7603495

[19] Zhang H, Liu S, Tang L, et al. Long non-coding RNA (LncRNA) MRPL23-AS1 promotes tumor progression and carcinogenesis in osteosarcoma by activating Wnt/β-catenin signaling via inhibiting microRNA miR-30b and upregulating myosin heavy chain 9 (MYH9). Bioengineered. 2021;12(1):162–171. 33356805 10.1080/21655979.2020.1863014 PMC8806232

[20] Li X, Chen B, Chi D, et al. lncRNA CASC9 regulates cell migration and invasion in hemangioma endothelial cells by targeting miR-125a-3p/Nrg1. Onco Targets Ther. 2019;12:423–432. 30662268 10.2147/OTT.S181914 PMC6327889

[21] Shao G, Wang M, Fan X, et al. lncRNA CASC9 positively regulates CHK1 to promote breast cancer cell proliferation and survival through sponging the miR‑195/497 cluster. Int J Oncol. 2019;54(5):1665–1675. 30816435 10.3892/ijo.2019.4734 PMC6438439

[22] Yan M, Ye L, Feng X, et al. MicroRNA-590-3p inhibits invasion and metastasis in triple-negative breast cancer by targeting Slug. Am J Cancer Res. 2020;10(3):965–974. 32266103 PMC7136920

[23] Abdolvahabi Z, Nourbakhsh M, Hosseinkhani S, et al. MicroRNA-590-3P suppresses cell survival and triggers breast cancer cell apoptosis via targeting sirtuin-1 and deacetylation of p53. J Cell Biochem. 2019;120(6):9356–9368. 30520099 10.1002/jcb.28211

[24] Wang WT, Qi Q, Zhao P, et al. miR-590-3p is a novel microRNA which suppresses osteosarcoma progression by targeting SOX9. Biomed Pharmacother. 2018;107:1763–1769. 30257395 10.1016/j.biopha.2018.06.124

[25] Gao J, Yu SR, Yuan Y, et al. MicroRNA-590-5p functions as a tumor suppressor in breast cancer conferring inhibitory effects on cell migration, invasion, and epithelial-mesenchymal transition by downregulating the Wnt-β-catenin signaling pathway. J Cell Physiol. 2019;234(2):1827–1841. 30191949 10.1002/jcp.27056

[26] Guan H, Liu J, Lv P, et al. MicroRNA‑590 inhibits migration, invasion and epithelial‑to‑mesenchymal transition of esophageal squamous cell carcinoma by targeting low‑density lipoprotein receptor‑related protein 6. Oncol Rep. 2020;44(4):1385–1392. 32945478 10.3892/or.2020.7692 PMC7448422

[27] Zhang Y, Chang J, Jiang W, et al. Long non-coding RNA CASC9/microRNA-590-3p axis participates in lutein-mediated suppression of breast cancer cell proliferation. Oncol Lett. 2021;22(1):544. 34084220 10.3892/ol.2021.12805 PMC8161424

[28] Yang C, Xu W, Gong J, et al. Six1 overexpression promotes glucose metabolism and invasion through regulation of GLUT3, MMP2 and snail in thyroid cancer cells. Onco Targets Ther. 2020;13:4855–4863. 32581547 10.2147/OTT.S227291 PMC7269010

[29] Kong D, Li A, Liu Y, et al. SIX1 activates STAT3 signaling to promote the proliferation of thyroid carcinoma via EYA1. Front Oncol. 2019;9:1450. 31921695 10.3389/fonc.2019.01450 PMC6933607

[30] Tang D, Zhao L, Peng C, et al. CRNDE promotes hepatocellular carcinoma progression by upregulating SIX1 through modulating miR-337-3p. J Cell Biochem. 2019;120(9):16128–16142. 31099050 10.1002/jcb.28894

[31] Smith AL, Iwanaga R, Drasin DJ, et al. The miR-106b-25 cluster targets Smad7, activates TGF-β signaling, and induces EMT and tumor initiating cell characteristics downstream of Six1 in human breast cancer. Oncogene. 2012;31(50):5162–5171. 22286770 10.1038/onc.2012.11 PMC3342483

[32] Micalizzi DS, Wang CA, Farabaugh SM, et al. Homeoprotein Six1 increases TGF-beta type I receptor and converts TGF-beta signaling from suppressive to supportive for tumor growth. Cancer Res. 2010;70(24):10371–10380. 21056993 10.1158/0008-5472.CAN-10-1354 PMC3072046

[33] Wan J, Yang J, Qiao C, et al. MicroRNA-362 inhibits cell proliferation and invasion by directly targeting SIX1 in colorectal cancer. Yonsei Med J. 2019;60(5):414–422. 31016902 10.3349/ymj.2019.60.5.414 PMC6479121

[34] Liu X, Zhou X, Chen Y, et al. miR-186-5p targeting SIX1 inhibits cisplatin resistance in non-small-cell lung cancer cells (NSCLCs). Neoplasma. 2020;67(1):147–157. 31686523 10.4149/neo_2019_190511N420

[35] Lv DQ, Li HY, Wu XM, et al. MiR-188 inhibits proliferation and promotes apoptosis of lung adenocarcinoma cells by targeting SIX1 to negatively regulate ERK signaling pathway. Eur Rev Med Pharmacol Sci. 2020;24(2):721–727. 32016974 10.26355/eurrev_202001_20051

[36] Yang ZC, Yi MJ, Shan YC, et al. Targeted inhibition of Six1 attenuates allergic airway inflammation and remodeling in asthmatic mice. Biomed Pharmacother. 2016;84:1820–1825. 27847210 10.1016/j.biopha.2016.10.090

[37] Du P, Luo K, Li G, et al. Long non-coding RNA VCAN-AS1 promotes the malignant behaviors of breast cancer by regulating the miR-106a-5p-mediated STAT3/HIF-1α pathway. Bioengineered. 2021;12(1):5028–5044. 34365889 10.1080/21655979.2021.1960774 PMC8806652

[38] Yin D, Lu X. Silencing of long non-coding RNA HCP5 inhibits proliferation, invasion, migration, and promotes apoptosis via regulation of miR-299-3p/SMAD5 axis in gastric cancer cells. Bioengineered. 2021;12(1):225–239. 33371778 10.1080/21655979.2020.1863619 PMC8806318

[39] Dong Y, Zhang X, Wang F, et al. [Construction of lentivirus plasmid pCDH-NLRX1 and stable expression of NLRX1 in A549 cells]. Xi Bao Yu Fen Zi Mian Yi Xue Za Zhi. 2020;36(2):152–156. 32314713

[40] Wu N, Zhang X, Bao Y, et al. Down-regulation of GAS5 ameliorates myocardial ischaemia/reperfusion injury via the miR-335/ROCK1/AKT/GSK-3β axis. J Cell Mol Med. 2019;23(12):8420–8431. 31625671 10.1111/jcmm.14724 PMC6850918

[41] Tang Z, Li C, Kang B, et al. GEPIA: a web server for cancer and normal gene expression profiling and interactive analyses. Nucleic Acids Res. 2017;45(W1):W98–W102. 28407145 10.1093/nar/gkx247 PMC5570223

[42] Paraskevopoulou MD, Vlachos IS, Karagkouni D, et al. DIANA-LncBase v2: indexing microRNA targets on non-coding transcripts. Nucleic Acids Res. 2016;44(D1):D231–8. 26612864 10.1093/nar/gkv1270 PMC4702897

[43] Agarwal V, Bell GW, Nam JW, et al. Predicting effective microRNA target sites in mammalian mRNAs. Elife. 2015;4:e05005. 10.7554/eLife.05005 PMC4532895 26267216

[44] Li Q, Li Z, Fan Z, et al. Involvement of non‑coding RNAs in the pathogenesis of myocardial ischemia/reperfusion injury (Review). Int J Mol Med. 2021;47(4):1. 10.3892/ijmm.2021.4875 PMC7895537 33576444

[45] Guan H, Shang G, Cui Y, et al. Long noncoding RNA APTR contributes to osteosarcoma progression through repression of miR-132-3p and upregulation of yes-associated protein 1. J Cell Physiol. 2019;234(6):8998–9007. 30317613 10.1002/jcp.27572

[46] Shang G, Wang Y, Xu Y, et al. Long non-coding RNA TCONS_00041960 enhances osteogenesis and inhibits adipogenesis of rat bone marrow mesenchymal stem cell by targeting miR-204-5p and miR-125a-3p. J Cell Physiol. 2018;233(8):6041–6051. 29319166 10.1002/jcp.26424 PMC5947671

[47] Nilsson F, Storm P, Sozzi E, et al. Profiling of coding and noncoding genes in human dopamine neuron differentiation. Cells. 2021;10(1):137. 33445654 10.3390/cells10010137 PMC7827700

[48] Yan S, Xu J, Liu B, et al. Long non-coding RNA BCAR4 aggravated proliferation and migration in esophageal squamous cell carcinoma by negatively regulating p53/p21 signaling pathway. Bioengineered. 2021;12(1):682–696. 33602031 10.1080/21655979.2021.1887645 PMC8291806

[49] Guan H, Mei Y, Mi Y, et al. Downregulation of lncRNA ANRIL suppresses growth and metastasis in human osteosarcoma cells. Onco Targets Ther. 2018;11:4893–4899. 30147340 10.2147/OTT.S170293 PMC6098425

[50] Sang Y, Tang J, Li S, et al. LncRNA PANDAR regulates the G1/S transition of breast cancer cells by suppressing p16(INK4A) expression. Sci Rep. 2016;6:22366. 26927017 10.1038/srep22366 PMC4772134

[51] Zhang Z, Chen F, Zhan H, et al. lncRNA CASC9 sponges miR‑758‑3p to promote proliferation and EMT in bladder cancer by upregulating TGF‑β2. Oncol Rep. 2021;45(1):265–277. 33200222 10.3892/or.2020.7852 PMC7716708

[52] Chen Y, Li Y, Gao H. Long noncoding RNA CASC9 promotes the proliferation and metastasis of papillary thyroid cancer via sponging miR-488-3p. Cancer Med. 2020;9(5):1830–1841. 31943867 10.1002/cam4.2839 PMC7050070

[53] Zhang J, Wang Q, Quan Z. Long non-coding RNA CASC9 enhances breast cancer progression by promoting metastasis through the meditation of miR-215/TWIST2 signaling associated with TGF-β expression. Biochem Biophys Res Commun. 2019;515(4):644–650. 31178137 10.1016/j.bbrc.2019.05.080

[54] Gao W, Gao J, Chen L, et al. XIST induced apoptosis of human osteosarcoma cells by activation of NF-kB/PUMA signal. Bioengineered. 2019;10(1):261–270. 31189404 10.1080/21655979.2019.1631104 PMC6592364

[55] Yang X, Zhang Q, Lu H, et al. Suppression of lncRNA MALAT1 Reduces LPS- or IL-17A-induced inflammatory response in human middle ear epithelial cells via the NF-κB signaling pathway. Biomed Res Int. 2021;2021:8844119. 33506040 10.1155/2021/8844119 PMC7808845

[56] Han B, Ge Y, Cui J, et al. Down-regulation of lncRNA DNAJC3-AS1 inhibits colon cancer via regulating miR-214-3p/LIVIN axis. Bioengineered. 2020;11(1):524–535. 32352854 10.1080/21655979.2020.1757224 PMC7202691

